# Impact of moderate to severe obstructive sleep apnea on the cognition in idiopathic pulmonary fibrosis

**DOI:** 10.1371/journal.pone.0211455

**Published:** 2019-02-01

**Authors:** Voicu Tudorache, Daniel Traila, Monica Marc, Cristian Oancea, Diana Manolescu, Emanuela Tudorache, Bogdan Timar, Alin Albai, Ovidiu Fira-Mladinescu

**Affiliations:** 1 Department of Pulmonology, “Victor Babes” University of Medicine and Pharmacy, Timişoara, Romania; 2 Department of Radiology, “Victor Babes” University of Medicine and Pharmacy, Timişoara, Romania; 3 Department of Functional Sciences, “Victor Babes” University of Medicine and Pharmacy, Timişoara, Romania; 4 Department of Internal Medicine, “Victor Babes” University of Medicine and Pharmacy, Timişoara, Romania; University of Rome Tor Vergata, ITALY

## Abstract

**Introduction:**

Idiopathic pulmonary fibrosis (IPF) is a relentlessly progressive lung disease with a fatal prognosis to whose rapid evolution multiple comorbidities may contribute, one of the most common being obstructive sleep apnea (OSA). There are several potential factors and conditions for the emergence of a cognitive deficit in relation to IPF or associated morbidities.

**Objectives:**

The goals of this study were to assess cognition in patients with IPF in stable phase and to identify clinical cognition modifiers.

**Methods:**

In a cross-sectional study, 23 patients with IPF were evaluated using Montreal Cognitive Assessment (MoCA), an instrument for detecting mild cognitive impairments and were screened for OSA through overnight cardiorespiratory polygraphy and for anxiety and depression with three specific scale (Generalized Anxiety Disorder 7-item scale: GAD-7; the Patient Health Questionnaire: PHQ-9; Hospital Anxiety and Depression Scale: HADS).

**Results:**

MoCA score was lower in patients with IPF when compared to controls subjects (24 [21,26] vs. 27 [26,28], p = 0.003) but not as significantly as in COPD patients (21 [18.8,23.3], p<0.0001). OSA was diagnosed in 19 (82.6%) IPF patients, 12 patients showed the presence of moderate-severe forms (63.15%). IPF patients with cognitive impairment (MoCA<23) exhibit a higher severity of OSA (apneea hypopnea index–AHI: 33.0±19.1 vs. 12.44±8.2, p = 0.018), and a higher Epworth score (7.1±3.3 vs. 4.3±1.8, p = 0.013). Anxiety and depression scores were not correlated with MoCA results.

**Conclusions:**

Impaired cognition in patients with IPF is mild and affect the areas of visuospatial abilities, language and working memory. OSA could be a possible predictor of IPF cognition deficit. Given the high prevalence of multiple types of sleep disorders in IPF patients, these should be investigated at least by cardiorespiratory polygraphy.

## Introduction

Idiopathic pulmonary fibrosis (IPF) is a *relentlessly* progressive lung disease with a *prognosis* that can be worse than many cancer forms. Having a median survival time of 2.5 to 3.5 years after diagnosis [1], IPF portends substantial morbidity and mortality outcomes, not all of which are directly related to the progressive fibrotic disease itself.

This older population with a median age of 66 years at diagnosis frequently experience various comorbidities which influence the clinical spectrum, progression and mortality of the disease. A recent retrospective IPF study found that 12% had no comorbidities, 58% had one to three comorbidities, and 30% had four to seven comorbidities [2]. Respiratory comorbidities, including emphysema (8%-34%), obstructive sleep apnea (58%-88%), lung cancer (3%-22%) and pulmonary hypertension (3%-84%) were common in many studies, although estimates vary widely depending on the source population. Nonrespiratory comorbidities such as gastro-esophageal reflux (30–80%), systemic arterial hypertension (14%-71%), ischaemic heart disease (4–68%), diabetes mellitus type 2 (10–33%) and depression (12–49%) are also highly prevalent [3,4].

IPF patients develop progressive ventilatory restriction and exercise intolerance. Alteration of blood gases is a common feature in IPF pathophysiology. Patients have transient or sustained hypoxia during sleep, accumulating a significant amount of time with SpO_2_ below 90% (an average of 50% of total sleep time) [5].

Nocturnal hypoxia is common in chronic fibrotic interstitial lung diseases, both in patients with obstructive sleep apnea (OSA) and in those without OSA simultaneously. This effect of decreasing lung volume during sleep may be even more important in supine position in patients with restrictive disorders such as IPF [6,7].

In addition to respiratory difficulties, chronic cough, medications, marked fatigue and tiredness, depression,contribute to the degradation of the quality of life [8,9].

Hypoxemia, a history of smoking, aging and chronic evolution are potential elements for the emergence of a cognitive deficit. Cognition has been explored by numerous studies in COPD [10], a condition in which all these factors are present and thus considered by some authorsa model of study for cognitive impairments secondary to chronic hypoxia due to lung disease[11,12].In IPF, a disease with a potentially rapid and frequent development of hypoxemia, it is surprising that cognitive impairment has been investigated in very few studies.Their results shows that almost half of IPF patients demonstrate at least mild cognitive dysfunction [13] and the cognitive decline corelates with IPF severity [14].

The morbidity associated with IPF has a wide and profound impact on the patients’ quality of life. OSA, a highly prevalent comorbidity in IPF, is a pathological condition in which cognitive impairment is well documented. However, from our knowledge there are no studies that have analyzed the OSA’s contribution to cognitive alteration in IPF.Therefore, cognition level and other patient-centered outcomes are important goals that should be evaluated in clinical research and practice. For IPF we do not currently have a specific cognitive assessment tool, thus researchers have used validated tools in cognition analysis of other chronic respiratory diseases. The potential problem is that these tools cannot capture many of the effects of IPF on patients' lives.

The **aim** of this study were to assess cognition in patients with IPF in stable phase and to identify clinical cognition modifiers.

## Materials and methods

### Study patients

This was a single-center, cross-sectional, descriptive study. IPF patients were identified from the clinical database of the Clinical Hospital of Infectious Disease and Pneumophtisiology ‘‘Dr. Victor Babes” Timisoara. After multidisciplinary review of the cases according to the ATS/ERS/JRS/ALAT 2013 guideline [15] the diagnosis of IPF was confirmed in 23 patients, either by the presence of a UIP pattern on HRCT in the appropriate clinical setting (20 patients), or by a combination of HRCT and characteristic findings on surgical lung biopsy (3 patients). The control grup consisted of 17 healthy volunteers between the ages of 40 and 80.

30 COPD patients were enrolled in the study as the comparison group. The inclusion criteria for COPD patients was GOLD grade 4 airflow severity, no use of long-term oxygen treatment (LTOT) and no exacerbation in the past two months.

Exclusion criteria for all groups were: alcohol consumption, neurological or psychiatric disorders, serious or unstable associated chronic diseases.

The study was approved by the ethics board of the Clinical Hospital of Infectious Disease and Pneumophtisiology ‘‘Dr. Victor Babes” Timisoara. Written informed consent was obtained from each patient.

### Measurements

Demographic data, smoking history and disease duration were collected in all subjects. All comorbidities were registered based on information from medical records. All IPF patients were screened for OSA through overnight cardiorespiratory polygraphy.

Cognition was evaluated using Montreal Cognitive Assessment (MoCA), a screening instrument with high specificity and sensitivity for detecting early cognitive impairments. MoCA evaluates several cognitive domains (short-term memory, visuospatial abilities, executive functioning, attention, concentration and working memory, language, orientation in time and space) to differentiate healthy cognitive aging from mild cognitive impairment (MCI) [16]. MoCA cutoff score considered to be indicative of cognitive impairment was 23, as this value has been found to have an excellent sensitivity and specificity (0.96 and 0.95, respectively) in previous studies [17,18].

All patients underwent pulmonary function tests and recording of O_2_ saturation (SpO_2_) by noninvasive pulse oximetry. Spirometry and assessment of the carbon monoxide diffusing capacity of the lung (DLco) were performed using standardized procedures [19,20]. To summarize the clinical-functional severity in IPF patients we used the GAP index, a multidimensional prognostic staging system based on clinical and physiologic variables (gender, age, FVC and DLco) [21].

Overnight cardiorespiratory polygraphy was performed using Porti 7 system that included thermistor sensor for breathing, thoracic wall motion to measure respiratory effort, oximetry to measure oxygen desaturations and a microphone for snoring. An apnea was defined as a cessation of airflow for 10 s and hypopnea was defined as a clear amplitude reduction of 50 to 90% in the thermistor transducer channel during sleep that was associated with an oxygen desaturation of 3%. OSA was defined as an apnea hypopnea index (AHI) over 5 events per hour. Whenever possible, polygraphy was performed without nasal oxygen except in those patients who were clinically judged to depend on oxygen supply. The subjects were asked to complete the Epworth sleepiness scale (ESS) which is a validated questionnaire commonly used for measuring daytime sleepiness.

Screening and measuring the severity of anxiety and depression were performed using Generalized Anxiety Disorder 7-item (GAD-7) scale (a seven-item, self-report anxiety questionnaire designed to assess the patient’s health status during the previous 2 weeks), the Patient Health Questionnaire (PHQ-9) (9-question depression scale) and Hospital Anxiety and Depression Scale (HADS) (a fourteen item scale that generates ordinal data, seven of the items relate to anxiety and seven relate to depression).

### Statistical analysis

Data were collected and analyzed using GraphPad Prism 7 and were presented as mean ± standard deviations in case of continuous variables with Gaussian distribution, median and interquartile range for continuous variables without Gaussian distribution or percentages for categorical variables.

To assess the significance of the differences between groups, the ANOVA along with Bonferroni post-hoc (means, Gaussian populations), Kruskal-Wallis and Mann-Whitney-U (medians, non-Gaussian populations) tests were used. Continuous variables distributions were tested for normality using Shapiro-Wilk test (if the p value was higher than 0.05, Gaussian distribution was assumed). The strength of correlations between variables was assessed using Spearman’s correlation coefficient and its statistical significance with t-distribution test.

A p-value < 0.05 was considered the threshold for statistical significance.

## Results

### Patient characteristics

The clinical characteristics and pulmonary function test results of patients are shown in Table 1. IPF patients were significantly older (67.6 years) compared to COPD patients (59.7 years), (p = 0.0036). COPD patients had a significantly longer duration of the disease (10.1 years) compared to IPF patients (3.9 years). Most IPF patients had moderate-severe GAP scores (39.1% GAP II and 17.4% GAP III). Twelve patients (52.2%) received antifibrotic treatment and 13 patients (56.5%) used LTOT.

**Table 1:** Clinical characteristics and physiology of patients.

Variable	Controls (No. = 17)	IPF (No. = 23)	COPD (No. = 30)
**Age** *(years)*^a^	60.6 ± 7.3	67.6 ± 8.7	59.7 ± 6.9
**Gender** *male (%*, *No*.*)*	52.9 (9)	56.5 (13)	73.3 (22)
**Body Mass Index** (*kg/m*^*2*^*)*^a^	27.3 ± 3.3	27.7 ± 4.5	25.8 ± 5.7
**Smoking history**			
Ever smoked *(%*, *No*.*)*	58.8 (10)	52.2 (12)	56.7 (17)
Pack-years^a^	32.4 ± 10.3	30.3 ± 12.1	33.5 ± 14.6
**SpO**_**2**_^a^	98.4 ± 4.1	92.4 ± 4.1	93.6 ± 3.7
**Pulmonary function**			
FVC *(% pred)*^a^	96.0 ± 7.3	71.3 ± 16.4	48.8 ± 15.1
FEV1 *(% pred)*^a^	95.0 ± 8.0	75.0 ± 15.9	23.7 ± 5.2
FEV1/FVC *(%)*^a^	97.6 ± 1.1	84.1 ± 5.5	38.2 ± 10.0
DLco*(% pred)*^a^	-	44.1 ± 13.9	54.2 ± 7.8
**Disease duration** *(years)*^a^	NA	3.9 ± 3.1	10.1 ± 2.0
**Disease severity**	NA	*GAP index*^b^ 4 [3, 5]	*Airflow severity* GOLD 4

^a^ Results are presented as mean ± standard deviation.

^b^ Results are presented as median and interquartile range.

NA: Not Applicable; SpO_2_: peripheral oxygen saturation; FVC: forced vital capacity; FEV1: forced expiratory volume in one second; DLco: diffusing capacity of lung for carbon monoxide; GAP: *Gender*, *Age*, *Physiology*Index; GOLD: the global initiative for chronic obstructive lung disease guidelines.

### Cognitive evaluation

**Fig 1:**
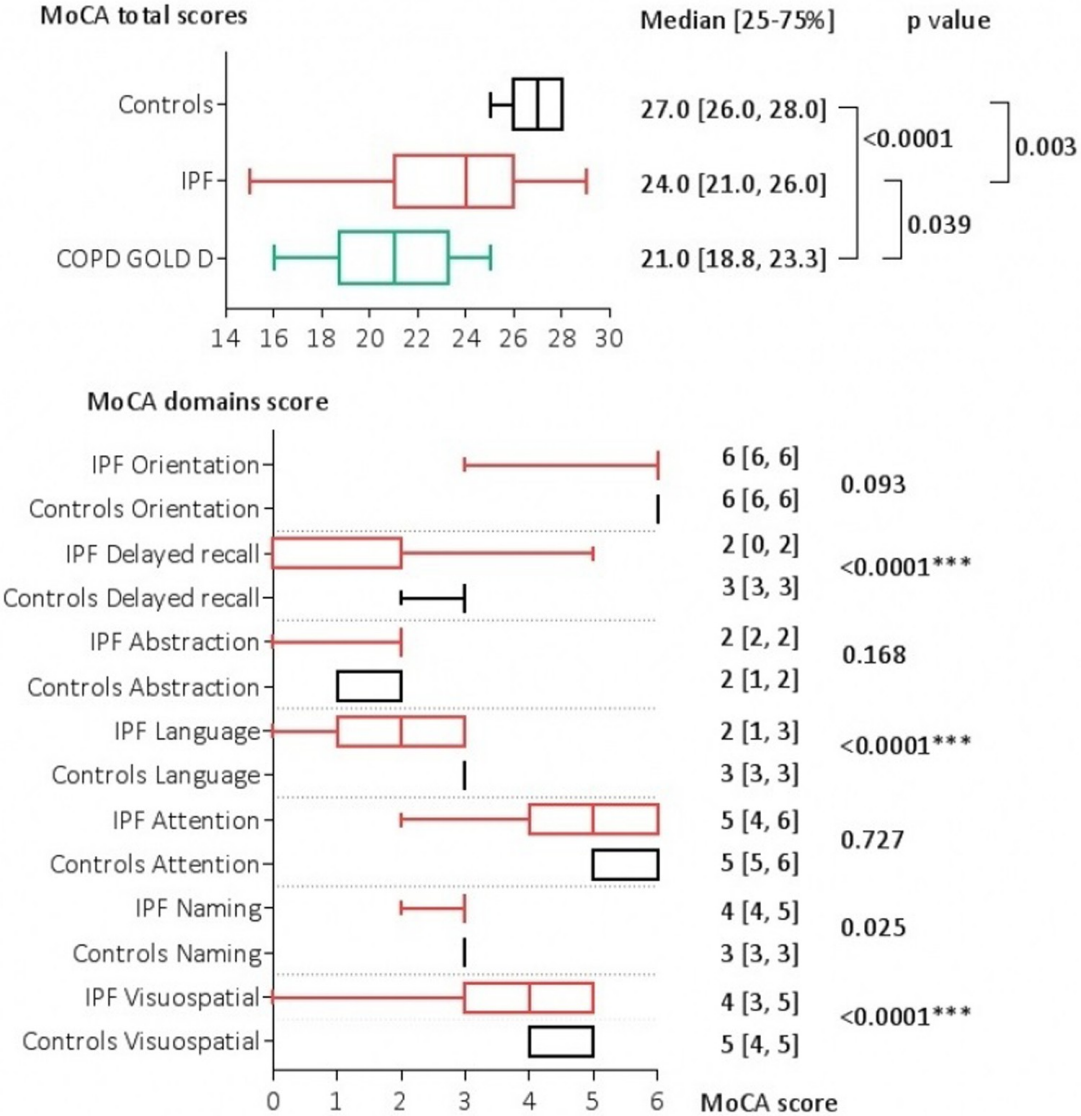
BoxPlot for MoCA in study subgroups.

MoCAglobal score was significantly lower for both IPF (24.0 [21.0, 26.0] and COPD patients (21.0 [18.8, 23.3]) when compared to controls (27.0 [26.0, 28.0]) (p<0.0001) (Fig 1). IPF patients presented mild impaired cognition due to significant lower scores for visuospatial (p<0.0115), language (p<0.0001) and delayed recall domains (p<0.0001). There was no significant correlation between cognitive function (MoCA scores) and demography, physiology, anxiety or depression in IPF patients.

COPD patients presented the most severe cognitive impairment. When compared to IPF, the decrease trend in MoCA score for COPD group was significant for two domains of the questionnaire: language (p<0.0001) and abstraction (p<0.0001), while the differences regarding orientation, delayed recall, attention, naming and visuospatial score had no statistical significance.

IPF patients had an average of 2.04 ± 0.88 associated comorbidities. These were in order of frequency: essential hypertension (43.5%), pulmonary hypertension (30.4%),emphysema (30.4%), ischemic heart disease (26.1%), gastroesophageal reflux disease (26.1%), heart failure %), diabetes mellitus (17.4%), dyslipidemia (8.7%), peripheral arterial disease (8.7%) and arrhythmias (8.7%).

**Fig 2:**
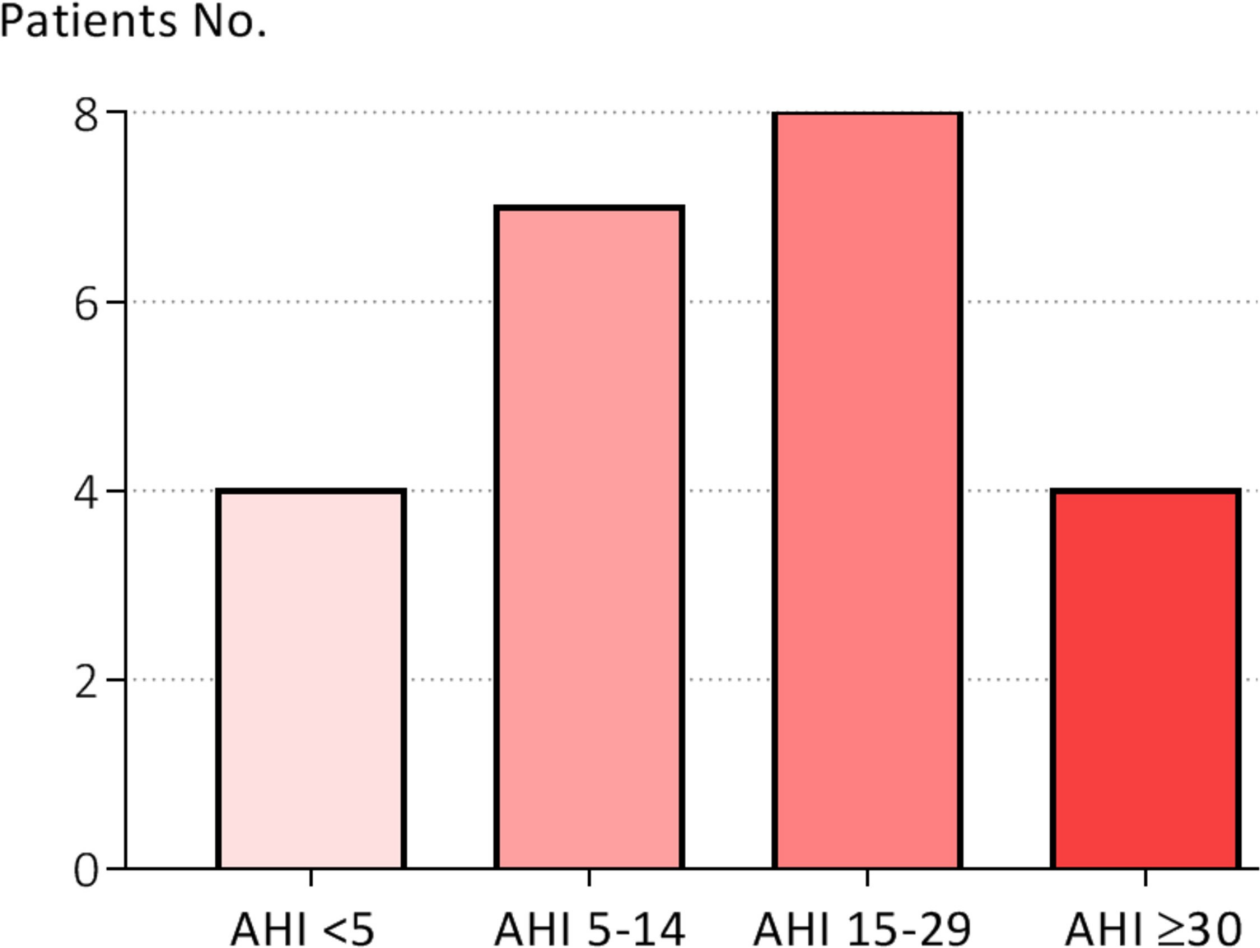
Prevalence and severity of OSA in IPF patients.

OSA was diagnosed in 19 (82.6%) IPF patients. The severity distribution (Fig 2) showed the presence of moderate-severe forms in 12 (63.15%) OSA patients.

IPF patients were stratified based on the MoCAcutoff score 23. The analysis of the two subgroups (Table 2) revealed statistically significant differences of the AHI and the Epworth questionnaire score. Thus, patients with cognitive impairment (MoCA<23) exhibit a higher severity of OSA (AHI 33.0 vs. 12.44, p = 0.018), and a higher Epworth score (7.1 vs. 4.3, p = 0.013). Demographic, physiology and anxiety and depression scores did not show significant differences between the two subgroups.

**Table 2:** Clinical characteristics of IPF patients stratified according to cognitive impairment.

Variable	MoCA< 23 (No. = 8)	MoCA ≥ 23 (No. = 15)	*P value*
**Age** *(years)*^a^	68.1 ± 8.5	67.3 ± 9.1	*0*.*837*
**BMI** *(kg/m*^*2*^*)*^a^	29.0 ± 3.1	27.0 ± 5.0	*0*.*240*
**FVC** *(% pred)*^a^	73.1 ± 13.2	70.3 ± 18.3	*0*.*674*
**DLco***(% pred)*^a^	45.8 ± 15.0	43.2 ± 13.7	*0*.*692*
**GAP index**^b^	3 [2.25, 4.5]	4 [3, 5]	*0*.*220*
**AHI**^a^	33.0 ± 19.1	12.4 ± 8.2	***0*.*018***
**ESS**^b^	6.5 [4.25, 8.75]	3 [3, 6]	***0*.*013***
**Min Saturation** *(%)*^a^	66.1 ± 13.7	69.4 ± 13.5	*0*.*645*
**Desaturation Index**^a^	26.38 ± 16.85	15.22 ± 8.39	*0*.*190*
**GAD-7**^b^	3 [2, 10.5]	3 [2, 7]	*0*.*884*
**PHQ-9**^b^	7 [3, 7.5]	3 [3, 6]	*0*.*933*
**HADS anxiety**^b^	9 [7.25, 9.75]	7 [5, 10]	*0*.*296*
**HADS depression**^b^	8.5 [5, 11.25]	7 [5, 10]	*0*.*715*

^a^ Results are presented as mean ± standard deviation.

^b^ Results are presented as median and interquartile range.

BMI: body mass index; FVC: forced vital capacity; DLco: diffusing capacity of lung for carbon monoxide; GAP: *Gender*, *Age*, *Physiology*Index; AHI: apneea hypopnea index; ESS: Epworth sleepiness scale; GAD-7: Generalized Anxiety Disorder 7-item; PHQ-9: Patient Health Questionnaire; HADS: Hospital Anxiety and Depression Scale.

## Discussion

Poor sleep quality is frequently met in IPF through sleep breathing disorders (SBDs) including OSA, implying increased sleep fragmentation, decreased slow wave and REM sleep, as well as sleep oxygen desaturation [5]. Our study found IPF association with OSA in 82.6% of patients and only a slight alteration of cognition (24 points on MoCA), which is consistent with other studies [13,22,23].

Sleep in IPF is a complex process that "mimics" the effects of maximum physical exercise. According to Kolilekas results, intermittent desaturation of sleep oxygen during the REM (rapid eyes movement) phase significantly exceeds maximum exercise and is associated with survival in patients with IPF [24]. Hypoxia, breathing difficulties and poor quality of life are also constantly found in COPD advanced stages. COPD, and in particular COPD associated with OSA, is a pathological condition which is accompanied by a significant deterioration of cognition [25]. Even in the absence of OSA, nocturnal hypoxemia could be the result of alveolar hypoventilation, ventilation-perfusion worsening ratio, and a desaturation tendency due to patients on the abrupt portion of the hemoglobin oxygen dissociation curve [26].

In our study although an increased AHI was present in most IPF patients it was not accompanied by a sustained and deep hypoxemia. Although it is known that hypoxemia will alter cognitive function, it is not completely clear what level of oxygenation is needed for proper cognitive function [27]. The mechanism for generating an adaptive response is governed by length of exposure and severity of hypoxia: acute, subacute or chronic and intermittent (obstructive sleep apnea syndrome—OSA) [28,29]. The duration of the illness may be important from the point of view of exposure to hypoxemia. In the IPF group, the duration was 3,9 years compared to 10,1 years among those with COPD, which may explain the more severe impairment of cognition in COPD patients.

Generally chronic diseases, as the disease progresses, develop debilitating features, and IPF is no exception. Cognitive impairment is multifactorial, but the history of smoking, aging and educational level are recognized as determinants [30,31]. In IPF there are many other potential factors: hypoxia, breathing difficulties and poor quality of life. In our study the severity of OSA was the single parameter correlated with cognitive deficits in patients with IPF. Although MoCA has not been specifically developed for use in IPF patients, its psychometric properties are appropriate and suggest that it may be useful to measure cognition in this patient population [32,33].Although at least 40 of psychometric tests in COPD have been described [10], MoCA is a sensitive instrument at discriminating mild cognitive impairment (MCI) from normal cognition and is validated in multiple settings and disorders.However a battery of complementary tests would have brought additional data.

Although daytime somnolence is one of the common symptoms in OSA, in patients with IPF and OSA, it is rare. They report more frequently excessive tiredness and fatigue.Other authors have shown that sleep disorders, although frequent and severe in patients with IPF are not associated with daytime sleepiness in more than 1 in 5 patients.[24]In our study, 82,6% of patients presented OSA without associating an increased Epworth score, which requires an active search for sleep disorders in this category of patients. In the literature, daytime somnolence in OSA patients is suggestive of hyperglycemia and hyperinsulinemia [34].

In a small study, with only 7 IPF patients undergoing pulmonary rehabilitation, Sprunger et al.[23],applying a battery of five psychometric tests, has shown impaired cognition only on the level of visual attention.In the study of Bors[14], there is a significant deterioration in cognition, but their cases were severe (Dlco = 30%pred), while ours were moderate (DLCO = 44%pred and GAP = 3 [2.25, 4.5]). A study developed by Sharp et al., applying MoCA, finds a mild impaired cognition in 46.7% of non-hypoxemic subjects with interstitial lung disease, much of it due to age; however the authors state "there remains a significant reduction in cognitive function in IPF" [13].

Although we applied three different scales to detect anxiety and depression, no correlations have been found. Depression alters the perception of respiratory symptoms, but at the same time it is a recognized factor that influences the level of cognition. This finding could be an additional explanation that patients with IPF have slightly altered cognition. This is in line with another recent study including 46 patients with moderate-severe IPF, in which depression scores were not suggestive of clinically meaningful depression [14]. DeVries et al. had previously found that while individuals with IPF may suffer from negative thoughts and feelings, they are not, in general, clinically depressed [35]. In the Australian IPF registry (516 cases), clinically significant depression assessed by HADS occurs only in 10.8% patients [36]. On the contrary, several studies have reported that the prevalence of depression ranges from 24.3±49.2%, while that of anxiety may be as high as 60%, in patients with IPF [37,38,39].

### Study limitations

(1) Relatively limited number of patients (IPF is a rare disease with some difficulty in recruiting large groups at a single center); (2) MoCA limitations in IPF since it was not originally developed for use in IPF patients. The potential problem is that this questionnaire may not capture many of the effects of IPF on patients lives; (3) Sleep-disordered breathing was not appreciated by polysomnography.

## Conclusions

We found a mild cognitive impairment in patients with IPF, that is related to the areas of visuospatial abilities, languageand working memory. In our opinion OSA could be a possible predictor of IPF cognition deficit.Given the high prevalence of multiple types of sleep disorders in all patients with IPF these should be investigated at least by cardiorespiratory polygraphy.

## Supporting information

S1 File IPF–study patients. (XLSX)

S2 File Control and COPD patients. (XLSX)
